# Role of Main Red Seaweed Bioactive Compounds in Modulating Redox Imbalance and Cholinergic Dysfunction: Insights from In Vitro Assays

**DOI:** 10.3390/cimb48020190

**Published:** 2026-02-07

**Authors:** João Ferreira, Mário Pacheco, Amélia M. Silva, Isabel Gaivão

**Affiliations:** 1 Animal and Veterinary Research Center (CECAV), Associate Laboratory for Animal and Veterinary Sciences (AL4AnimalS), University of Trás-os-Montes and Alto Douro (UTAD), 5000-801 Vila Real, Portugal; 2 Centre for Research and Technology of Agro-Environmental and Biological Sciences (CITAB), Inov4Agro, University of Trás-os-Montes and Alto Douro (UTAD), Quinta de Prados, 5000-801 Vila Real, Portugal; amsilva@utad.pt; 3 Centre for Environmental and Marine Studies (CESAM), Department of Biology, University of Aveiro, Campus Universitário de Santiago, 3810-193 Aveiro, Portugal; mpacheco@ua.pt; 4 Department of Biology and Environment (DeBA), Scholl of Life and Environmental Sciences (ECVA), University of Trás-os-Montes and Alto Douro (UTAD), 5000-801 Vila Real, Portugal; 5 Department of Genetics & Biotechnology (DGB), Scholl of Life and Environmental Sciences (ECVA), University of Trás-os-Montes and Alto Douro (UTAD), 5000-801 Vila Real, Portugal

**Keywords:** sulfated polysaccharides, mycosporine-like amino acids, bromophenols, ROS, RNS, antioxidant activity, anti-inflammatory activity, immunostimulatory activity, acetylcholinesterase inhibition, functional food

## Abstract

Oxidative and nitrosative stress are key contributors to the development and progression of chronic inflammatory disorders, cancer and neurodegenerative diseases (viz., Alzheimer’s disease). Cholinergic dysfunction is a major hallmark of Alzheimer’s disease and is closely associated with these processes. Red seaweeds are rich in bioactive compounds that have been increasingly investigated for their potential to modulate these processes. This review aims to examine the role of major red seaweed-derived metabolites in regulating redox imbalance, immunomodulatory capacity and acetylcholinesterase activity, with emphasis on in vitro studies. An analysis of peer-reviewed literature was conducted, focusing on chemical, biochemical and cell-based assays. Studies assessed antioxidant activity, anti-inflammatory and immunostimulatory effects, and acetylcholinesterase inhibition of isolated compounds/fractions of red seaweed using established methods, including radical scavenging assays, Griess-based nitrite assay and enzyme inhibition assays. Sulfated polysaccharides, oligosaccharides, mycosporine-like amino acids (MAAs), phycoerythrin, bromophenols, phlorotannin and terpenoid-derived metabolites demonstrated antioxidant capacity through radical scavenging, metal chelation and modulation of endogenous antioxidants. They also modulated inflammatory mediators, including nitric oxide and pro-inflammatory cytokines, and inhibited acetylcholinesterase (AChE) activity. In vitro evidence supports red seaweed-derived compounds as promising modulators of redox homeostasis, inflammation and cholinergic function, highlighting their relevance as functional food ingredients, while underscoring the need for in vivo and clinical validation.

## 1. Unique Chemical Composition of Red Seaweeds and Their Use as Food

Red seaweeds, classified within the phylum Rhodophyta, comprise the most extensive lineage of seaweeds under the kingdom Plantae [1]. This group encompasses roughly 7000 described species distributed throughout diverse marine ecosystems, from sunlit intertidal zones to deeper oceanic regions [2,3,4].

A defining feature of Rhodophyta lies in their chemical composition, which distinguishes them from the other algal phyla and land plants [5]. Although they contain chlorophyll along with carotenoids and xanthophylls, their distinctive pigmentation arises from phycobiliproteins, namely, phycocyanin and phycoerythrin [3]. Structurally, red seaweeds possess cell walls composed of unique sulfated polysaccharides intertwined with cellulose [4,6]. Among these high-molecular-weight polymers, agar and carrageenan consist of galactose residues supporting sulfate substituents and stand out for their abundance and structural complexity [3,7]. Agar mainly comprises agarose, accompanied by smaller amounts of agaropectin, while carrageenan occurs primarily as ι-, κ- and λ-types, differing in sulfation degree and the presence of 3,6-anhydrogalactose moieties [6,7]. Another distinctive chemical characteristic of red seaweeds is their ability to produce mycosporine-like amino acids (MAAs), which are low-molecular-weight, water-soluble compounds synthesized primarily in response to high solar radiation [8,9]. These metabolites are characterized by a cyclohexenone or cyclohexenimine ring conjugated to amino acids or amino alcohols [8,10]. Structural variations in these side chains underlie considerable molecular diversity among MAAs, leading to the identification of distinct types, including mycosporine–glycine, palythine, asterina-330, shinorine and porphyra-334 [8,9,10,11,12]. In addition to MAAs, some seaweed species synthesize other secondary metabolites, including phenolic compounds (such as bromophenols), terpenoids and alkaloids [3,13].

Because of their chemical richness, red seaweeds have long been integrated into human diets, mostly in East and Southeast Asia, traditionally consumed as vegetables, sushi wrappers and as ingredients in food products [4,14,15,16]. Beyond their direct consumption, red seaweeds are also used in the form of dietary supplements (isolated compounds or fractions) and infusions [17]. Moreover, agar and carrageenan are used as food additives, considering their gelling, thickening and stabilizing properties in several food products worldwide [3,18,19]. The growing global interest in seaweeds stems from cultural exchange, and the search for new products incorporating red seaweeds and its derivatives into the diet, whether as functional foods or in the form of nutraceuticals, constitutes a sustainable strategy for promoting human health, owing to its ability to modulate a wide range of physiological processes [5].

## 2. Red Seaweed Bioactive Compounds in Redox Regulation

### 2.1. Reactive Oxygen and Nitrogen Species and Disease

**Figure 1:**
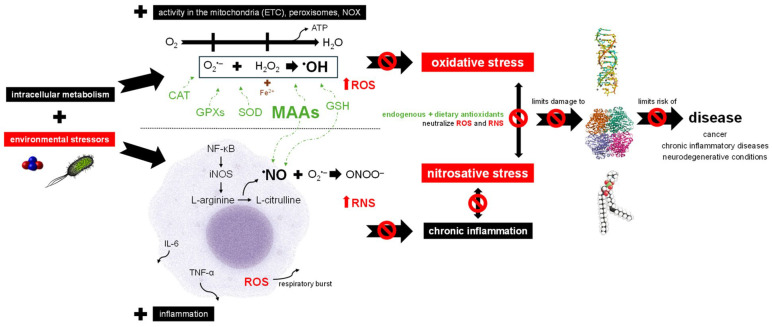
Impact of red seaweed-derived MAAs on redox balance and disease risk. The scheme provides a simplified representation of the complex processes and mediators involved. Environmental stressors include α-particles emitted during radon (Rn) decay and *Escherichia coli*. Mitochondria, through electron transport chain (ETC), generate reactive oxygen species (ROS) as they reduce O_2_ to H_2_O while producing energy in the form of ATP [21,22]. Although mitochondria are a major source, ROS are also produced in peroxisomes and by cytoplasmatic enzymes like NOX. Environmental stressors further exacerbate ROS formation. Key ROS include the superoxide radical (O_2_^•−^), hydrogen peroxide (H_2_O_2_) and the hydroxyl radical (^•^OH). The latter is formed through the Haber–Weiss reaction (interaction of O_2_^•−^ with H_2_O_2_) and the Fenton reaction mediated by heavy metals such as Fe^2+^ [23,24]. When endogenous enzymatic (e.g., superoxide dismutase (SOD), catalase (CAT), glutathione peroxidases (GPXs)) and non-enzymatic antioxidants (e.g., glutathione (GSH)) are supported with the neutralizing capacity of dietary antioxidants like MAAs, the capability of the organism to prevent oxidative stress increases [21,25,26]. As ROS and environmental stressors activate macrophages though the nuclear factor kappa B (NF-κB) signaling pathway, inflammation occurs [27]. NF-κB upregulates inducible nitric oxide synthase (iNOS), catalyzing the conversion of L-arginine into L-citrulline and releasing nitric oxide (NO). This process generates further reactive nitrogen species (RNS), such as peroxynitrite (ONOO^−^). Simultaneously, pro-inflammatory cytokines, including tumor necrosis factor-alpha (TNF-α) and interleukin-6 (IL-6) are secreted [6,27]. Macrophages also contribute to ROS production via NOX during respiratory bursts [20]. However, the broad arsenal of antioxidants can neutralize excessive levels of ROS and RNS through antioxidant and anti-inflammatory actions, thereby preventing chronic inflammation and the intertwined effects of oxidative and nitrosative stress, which would otherwise reinforce each other in a vicious cycle, leading to progressive damage to biomolecules [20,27,28,29,30]. Consequently, these compounds may ultimately contribute to a reduced risk of associated disease development and/or progression [21]. Abbreviations: ETC, electron transport chain; NOX, nicotinamide adenine dinucleotide phosphate oxidases; ATP, adenosine triphosphate; ROS, reactive oxygen species; O_2_^•−^, superoxide radical; H_2_O_2_, hydrogen peroxide; ^•^OH, hydroxyl radical; SOD, superoxide dismutase; CAT, catalase; GPXs, glutathione peroxidases; MAAs, mycosporine-like amino acids; GSH, glutathione; NF-κB, nuclear factor kappa B; iNOS, inducible nitric oxide synthase; NO, nitric oxide; RNS, reactive nitrogen species; ONOO^−^, peroxynitrite; TNF-α, tumor necrosis factor-alpha; IL-6, interleukin-6.

Oxygen acts as the final electron acceptor in the mitochondrial electron transport chain (ETC), representing the last step of cellular respiration (Figure 1). Upon receiving electrons and protons, O_2_ is reduced into H_2_O. This step is crucial for aerobic metabolism as it facilitates efficient adenosine triphosphate (ATP) production. However, electron leakage results in the generation of reactive oxygen species (ROS) as byproducts during this process, that act as signaling molecules, influencing various cellular processes. Beyond mitochondrial production, ROS can also be produced in peroxisomes and by cytoplasmic enzymes like the nicotinamide adenine dinucleotide phosphate (NADPH) oxidase (NOX) family as part of intracellular metabolism (Figure 1) [20,21,22]. In addition to endogenous sources, ROS production has been observed following the metabolism of exogenous toxic substances (Figure 1) [21].

ROS include both oxygen radicals (free radicals) such as the superoxide anion radical (O_2_^•−^) and the hydroxyl radical (^•^OH), as well as non-radicals that act as oxidative agents, like hydrogen peroxide (H_2_O_2_) (Figure 1). Significant problems occur when O_2_^•−^ and H_2_O_2_ react through the Haber–Weiss reaction, producing the highly toxic ^•^OH, the most damaging ROS to biological systems. Moreover, heavy metals such as Fe and Cu produce ^•^OH via the Fenton reaction where, as an example, ferrous Fe (Fe^2+^) adds an electron to H_2_O_2_, resulting in the production of ^•^OH (Figure 1) [20,23,24]. Beyond these, numerous other ROS contribute to the complexity of redox interactions in biological systems [21].

However, when excessive ROS accumulates in the body and cannot be neutralized by antioxidants, oxidative stress occurs. This imbalance, triggered by both endogenous and exogenous factors, enables ROS to damage macromolecules through direct interaction (Figure 1) [20,21,25]. Endogenous contributors include increased ROS generation due to mitochondrial dysfunction and/or impaired antioxidant defenses resulting from enzyme dysfunction and/or nutrient deficiencies. Exogenous sources encompass ionizing radiation, as well as exposure to environmental pollutants (Figure 1) [20,21]. Moreover, exogenous stressors can exacerbate mitochondrial dysfunction, thereby amplifying endogenous ROS production and intensifying oxidative stress. These environmental stressors can generate ROS directly or stimulate secondary responses such as inflammation, during which immune cells release ROS (Figure 1) as a defense strategy [20,31].

Apart from ROS, which are the most significant reactive species in biological systems, other reactive species include reactive nitrogen species (RNS), particularly nitric oxide (NO) and its derivatives (Figure 1) [21,32]. NO is produced by a family of enzymes known as nitric oxide synthases (NOS), the neuronal (nNOS or NOS-1, encoded by *NOS1* gene), the endothelial (eNOS or NOS-3, encoded by *NOS3*) and the inducible (iNOS or NOS-2, encoded by *NOS2*) isoforms [33]. In response to inflammatory signals, iNOS catalyzes the conversion of L-arginine into L-citrulline and NO in macrophages (Figure 1). While NO plays essential physiological roles, it is also a free radical that, when overproduced, reacts with O_2_^•−^ to generate additional RNS (Figure 1) [29,30,34]. Analogous to oxidative stress, nitrosative stress occurs when there is an imbalance between RNS levels and the body’s capacity to neutralize them (Figure 1) [20,32].

Inflammation is a key process of the innate immune system. Central to this process are macrophages, which play crucial roles in initiating, sustaining and resolving the inflammatory response (Figure 1) [34,35]. Pro-inflammatory stimuli, including pathogen-associated molecules (PAMPs) such as lipopolysaccharides (LPSs), activate macrophages primarily through the activation of TLRs (Toll-like receptors), commonly the TLR4, that activate the transcription factor nuclear factor kappa B (NF-κB) (Figure 1) and mitogen-activated protein kinases (MAPK) pathways [25,27,36,37]. NF-κB activation induces the expression of iNOS, resulting in NO production, and of various pro-inflammatory cytokines, such as tumor necrosis factor-alpha (TNF-α) and interleukin-6 (IL-6) (Figure 1) [37,38]. It also promotes cyclooxygenase-2 (COX-2) expression, which catalyzes the conversion of arachidonic acid to prostaglandins. These mediators collectively coordinate an inflammatory response aimed at combating pathological conditions, such as bacterial infections, but also products of cellular damage (Figure 1) [6,27,39]. When inflammation becomes persistent, often due to sustained levels of NO, ROS and other mediators, it progresses to chronic inflammation. This state is linked with nitrosative stress, continuous tissue damage and genomic instability, ultimately increasing the risk of associated diseases (Figure 1) [27,28].

Oxidative and nitrosative stress lead to significant damage to macromolecules (Figure 1) [25,29]. Such damage can disrupt the function of enzymes, transporters, signal transducers and structural proteins [20,21,23,24,25]. The oxidative degradation of lipids, lipid peroxidation, can produce a range of toxic byproducts, such as ketones, that can lead to cell dysfunction, apoptosis or necrosis [40]. Regarding nucleic acids, DNA is particularly vulnerable to radical-induced damage, which can cause substantial genetic alterations like strand breaks and base modifications [20,21,23,24,25]. The interplay between oxidative and nitrosative stress further exacerbates cellular damage and disease risk, being implicated in a range of diseases, including cardiovascular disorders, diabetes mellitus, hyperoxaluria, neurodegenerative conditions, depression, chronic inflammatory diseases and cancer (Figure 1) [20,21].

### 2.2. Red Seaweed Bioactive Compounds Promote Antioxidant Protection and Immunomodulation

Antioxidants act to reduce intracellular levels of ROS and RNS, particularly free radicals, thereby preventing or delaying damage to macromolecules (Figure 1). By counteracting oxidative and nitrosative stress, antioxidants contribute to lowering the risk of diseases associated with these processes, although additional factors also influence disease initiation and progression. Factors such as genetic predisposition, lifestyle choices and chronic inflammation can amplify oxidative stress and promote disease [25,30,41]. To avoid excessive ROS and RNS accumulation, the body relies on a coordinated antioxidant defense system composed of both enzymatic and non-enzymatic components (Figure 1). Despite their mechanistic diversity, the primary protective strategy of antioxidants is the scavenging and neutralization of ROS/RNS before they reach damaging levels [30,37,42].

Key antioxidant enzymes include superoxide dismutase (SOD), catalase (CAT) and glutathione peroxidases (GPXs) (Figure 1). SOD protects cells by scavenging O_2_^•−^ and converting it into H_2_O_2_; CAT then breaks down H_2_O_2_ into H_2_O, and similarly, GPXs reduce H_2_O_2_ using the reducing power of glutathione (GSH) [20,25,29,30]. Endogenous non-enzymatic antioxidants play a vital role in the body’s antioxidant defense system (Figure 1) [26,37,43]. Among these, the tripeptide GSH is particularly significant due to its role in supporting GPXs [20,29,42]. Moreover, GSH plays a critical role in buffering NO and converting it into *S*-nitrosoglutathione (GSNO) (Figure 1) [29]. Also, ferritin and lactoferrin are proteins that sequester Fe, reducing its availability for the Fenton reaction [20,44].

**Figure 2:**
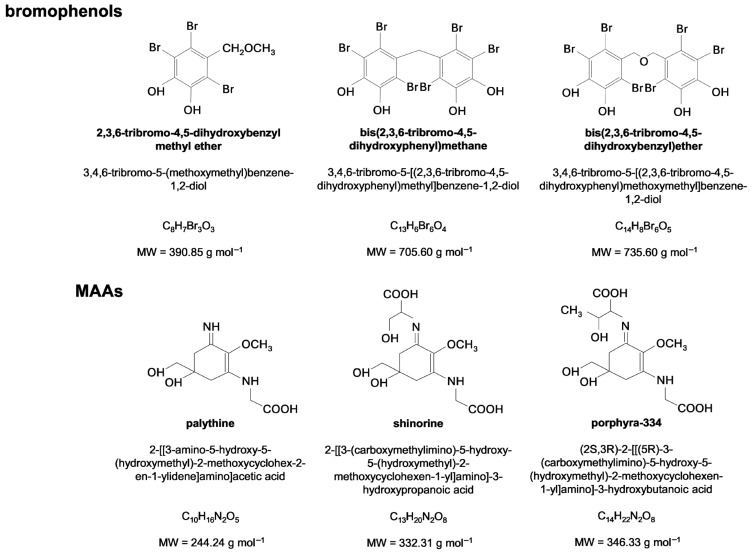
Chemical structure of the red seaweed-derived bromophenols and mycosporine-like amino acids (MAAs) presented in Table 1. The IUPAC names, molecular formulas and molecular weights are displayed for each compound. Data were retrieved from National Center for Biotechnology Information [56,57,58,59,60,61].

Dietary antioxidants are of great interest for their potential to complement the body’s endogenous antioxidant defenses (Figure 1). Similar to endogenous non-enzymatic antioxidants, dietary compounds counteract ROS and RNS through multiple mechanisms, including scavenging free radicals and donating electrons to facilitate ROS and RNS conversion, and chelating metal ions involved in the Fenton reaction [21,23,41]. They can also donate H^+^ to convert ^•^OH into H_2_O [23]. The antioxidant potential of crude and fractionated plant extracts is among the most extensively studied bioactivities, reflecting the critical role of oxidative stress in various pathological conditions [3,30,37]. Specifically, phenolic compounds, namely, bromophenols (Figure 2) and phlorotannin, MAAs (Figure 1 and Figure 2), terpenoids, phycoerythrin and sulfated polysaccharides extracted from several red seaweeds showed antioxidant potential, as demonstrated by numerous in vitro assays, including 2,2-diphenyl-1-picrylhydrazyl (DPPH^•^) radical, ferric reducing antioxidant power (FRAP), 2,2′-azino-bis(3-ethylbenzothiazoline-6-sulfonic acid) (ABTS^•+^) radical and oxygen radical absorbance capacity (ORAC) assays, which showed the capacity of these algal compounds to donate H^+^ or/and electrons, their hydroxyl radical (^•^OH) scavenging potential that showed their potential in scavenging ^•^OH directly, and the ferrous ion-chelating capacity assay, which demonstrated their ability to bind to metal ions (Table 1) [9,45,46,47,48,49,50,51,52,53,54,55].

Comparison with several red seaweed-derived antioxidants (Table 1) reveals that the bromophenol bis(2,3,6-tribromo-4,5-dihydroxyphenyl)methane (Figure 2) stands out as the most effective DPPH^•^ radical scavenger, displaying a remarkably low IC_50_ value of 0.0057 mg mL^−1^ [52]. This bromophenol compound is highly brominated and fully substituted, and each molecule contains two 2,3,6-tribromo-4,5-dihydroxyphenyl structural units linked by a methane group (Figure 2) [52]. The pronounced DPPH^•^ scavenging activity of bis(2,3,6-tribromo-4,5-dihydroxyphenyl)methane is most plausibly attributed to the presence of the hydroxyl groups (Figure 2) capable of quenching DPPH^•^ radicals through hydrogen atom transfer and/or electron transfer mechanisms [62].

**Table 1:** Isolated compounds and fractions extracted from red seaweeds and their antioxidant activities evaluated using different in vitro assays.

Seaweed Species	Isolated Compound/ Fraction	Type of Assay	Result	Dose	Reference
*Symphyocladia latiuscula*	Bromophenols	DPPH^•^ radical	IC_50_ = 0.006–0.011 mg mL^−1^ (9) ^a^	-	[52]
*Kappaphycus alvarezii*	Meroterpenoids	ABTS^•+^ radical	IC_50_ = 0.34, 0.58, 0.72 mg mL^−1^ (3) ^a^	-	[53]
		DPPH^•^ radical	IC_50_ = 0.31, 0.52, 0.70 mg mL^−1^ (3) ^a^	-	[53]
*Grateloupia elliptica*	Phlorotannin	DPPH^•^ radical	IC_50_ = 0.02 mg mL^−1^	-	[54]
*Kappaphycus alvarezii*	Phycoerythrin	ABTS^•+^ radical	62% (% control) ^b^	1.00 mg mL^−1^	[55]
		DPPH^•^ radical	58% (% control) ^b^	1.00 mg mL^−1^	[55]
		H_2_O_2_ scavenging	68% (% control) ^b^	1.00 mg mL^−1^	[55]
		Phosphomolybdenum	73% (% control) ^b^	1.00 mg mL^−1^	[55]
		Reducing power	71% (% control) ^b^	1.00 mg mL^−1^	[55]
*Gracilaria domingensis*	Palythine (MAAs)	ABTS^•+^ radical	46.6 mmol TE mol^−1^	-	[45]
		Ferrous ion-chelating	IC_50_ > 0.07 mg mL^−1^	-	[45]
		Folin–Ciocalteu	905.0 mmol TE mol^−1^	-	[45]
		FRAP	7.1 mmol TE mol^−1^	-	[45]
		ORAC	57.4 mmol TE mol^−1^	-	[45]
*Gracilaria domingensis*	Porphyra-334 (MAAs)	ABTS^•+^ radical	28.9 mmol TE mol^−1^	-	[45]
*Porphyra umbilicalis*			IC_50_ = 0.05 mg mL^−1^	-	[46]
*Porphyra yezoensis*		DPPH^•^ radical	IC_50_ = 0.06 mg mL^−1^	-	[9]
*Gracilaria domingensis*		Ferrous ion-chelating	IC_50_ > 0.07 mg mL^−1^	-	[45]
		Folin–Ciocalteu	1287.0 mmol TE mol^−1^	-	[45]
		FRAP	5.7 mmol TE mol^−1^	-	[45]
		ORAC	33.8 mmol TE mol^−1^	-	[45]
*Porphyra yezoensis*			51% (% Trolox) ^c^	0.035 mg mL^−1^	[9]
*Ahnfeltiopsis devoniensis*	Shinorine	ABTS^•+^ radical	IC_50_ = 0.03 mg mL^−1^	-	[47]
*Gracilaria domingensis*	(MAAs)		29.9 mmol TE mol^−1^	-	[45]
*Porphyra umbilicalis*			IC_50_ = 0.03 mg mL^−1^	-	[46]
*Gloiopeltis furcata*		DPPH^•^ radical	IC_50_ = 0.03 mg mL^−1^	-	[9]
*Gracilaria domingensis*		Ferrous ion-chelating	IC_50_ = 0.07 mg mL^−1^	-	[45]
		Folin–Ciocalteu	1032.0 mmol TE mol^−1^	-	[45]
		FRAP	8.5 mmol TE mol^−1^	-	[45]
		ORAC	75.5 mmol TE mol^−1^	-	[45]
*Gloiopeltis furcata*			17% (% Trolox) ^c^	0.033 mg mL^−1^	[9]
*Asparagopsis armata*	Sulfated	ABTS^•+^ radical	52% (% control) ^b^	1.00 mg mL^−1^	[49]
*Gelidium corneum*	polysaccharides		~12% (% control) ^b^	0.50 mg mL^−1^	[48]
*Gelidium pristoides*			71% (% control) ^b^	0.33 mg mL^−1^	[63]
*Gracilaria gracilis*			61% (% control) ^b^	0.33 mg mL^−1^	[64]
*Porphyra umbilicalis*			~20% (% control) ^b^	0.50 mg mL^−1^	[48]
*Gelidium pristoides*		DPPH^•^ radical	~38% (% control) ^b^	0.33 mg mL^−1^	[63]
*Gracilaria gracilis*			~30% (% control) ^b^	0.33 mg mL^−1^	[64]
*Gelidium pristoides*		Ferrous ion-chelating	62% (% control) ^b^	0.10 mg mL^−1^	[63]
*Gracilaria caudata*			0, 40, 70% (% control) ^b^ (3) ^a^	1.00 mg mL^−1^	[65]
*Gracilaria gracilis*			63% (% control) ^b^	0.10 mg mL^−1^	[64]
*Asparagopsis armata*		^•^OH scavenging	55% (% control) ^b^	5.00 mg mL^−1^	[49]
*Gelidium pristoides*			~50% (% control) ^b^	0.10 mg mL^−1^	[63]
*Gracilaria caudata*			~1, 30, 8% (% control) ^b^ (3) ^a^	1.00 mg mL^−1^	[65]
*Gracilaria gracilis*			52% (% control) ^b^	0.10 mg mL^−1^	[64]
*Porphyra haitanensis*			~23% (% control) ^b^	5.00 mg mL^−1^	[50]
*Asparagopsis armata*		Phosphomolybdenum	190.0 μmol mL α-TE^−1^	5.00 mg mL^−1^	[49]
*Gracilaria caudata*			53.0, 25.4, 63.9 mg AA g^−1^ (3) ^a^	-	[65]
		Reducing power	100, 100, 80% (% control) ^b^ (3) ^a^	1.00 mg mL^−1^	[65]
		O_2_^•−^ scavenging	~24, 54, 18% (% control) ^b^ (3) ^a^	0.05 mg mL^−1^	[65]

Abbreviations: AA, ascorbic acid; ABTS^•+^, 2,2′-azino-bis(3-ethylbenzothiazoline-6-sulfonic acid); DPPH^•^, 2,2-diphenyl-1-picrylhydrazyl (DPPH^•^); FRAP, ferric reducing antioxidant power; MAA, mycosporine-like amino acids; ORAC, oxygen radical absorbance capacity; TE, Trolox equivalents; α-TE, α-tocopherol equivalents. ^a^ number of compounds/fractions analyzed; ^b^ percentage relative to control (defined as 0% activity); ^c^ percentage relative to Trolox (defined as 100% activity).

Moreover, the relatively simple and sterically accessible molecular architecture of this compound is likely to facilitate efficient DPPH^•^ radical scavenging, consistent with the comparable activities reported in the same study for 2,3,6-tribromo-4,5-dihydroxybenzyl methyl ether (IC_50_ = 0.0061 mg mL^−1^) and bis-(2,3,6-tribromo-4,5-dihydroxybenzyl) ether (IC_50_ = 0.0063 mg mL^−1^) (Table 1 and Figure 2) [52]. In addition, notable antioxidant activity was also observed for MAAs, particularly shinorine (Figure 2), which demonstrated considerable radical scavenging capacity in this assay (Table 1) [9]. Conversely, the pigment phycoerythrin displayed a much higher IC_50_ value (approximately 0.86 mg mL^−1^), making it the least effective bioactive compound from red seaweeds tested for DPPH^•^ radical scavenging (Table 1) [64]. The lower activity observed for phycoerythrin (red-protein complex) reflects the limited suitability of the DPPH^•^ assay for protein-based antioxidants, whereas small compounds, such as bromophenols and MAAs (Figure 2), are inherently more reactive toward the DPPH^•^ radical [66].

Relative to the ABTS^•+^ radical scavenging assay, all MAAs evaluated exhibited strong antioxidant capacity, with palythine showing the highest activity (Table 1 and Figure 2) [45]. The ability of MAAs to quench ABTS^•+^ is largely attributed to electron transfer processes, likely influenced by the ionization of amine and carboxyl functional groups present in their molecular structures (Figure 2) [45]. This is consistent with reports indicating that small, highly polar antioxidant compounds display pronounced radical scavenging efficiency in the ABTS^•+^ assay [67,68]. In contrast to the DPPH^•^ assay, the ABTS^•+^ method is considered more versatile and suitable for assessing the antioxidant capacity of a broad range of compounds and food matrices, particularly hydrophilic antioxidants [67,68]. Contrarily, the sulfated polysaccharide fractions reported by Díaz et al. [48] exhibited comparatively low antioxidant activity in this assay, which may be attributed to its high molecular weight and structural complexity, factors known to limit the accessibility and reactivity of functional groups toward radical species. Nonetheless, sulfated polysaccharide fractions reported by Olasehinde et al. [63,64] demonstrated strong ferrous ion-chelating activity, highlighting their potential antioxidant action through metal chelation rather than direct radical scavenging.

Furthermore, the MAAs palythine, porphyra-334 and shinorine (Figure 2) exhibited the highest antioxidant capacity among the compounds and fractions evaluated in the ferrous ion-chelating, Folin–Ciocalteu and FRAP assays (Table 1) [9,45]. In the ferrous ion-chelating assay, MAAs were able to complex Fe^2+^ possibly through their nitrogen- and oxygen-containing functional groups, thereby limiting radical generation via Fenton reaction [45]. In the Folin–Ciocalteu and FRAP assays, as for ABTS^•+^ assay, MAAs act as effective reducing agents through electron transfer mechanisms [45]. In MAAs, the central chromophore contains an imine functionality, whereas amine and carboxyl groups are located in the amino acid-derived side chains (Figure 2) [45]. Together, these features are associated with electron transfer processes and may contribute, particularly through nitrogen-containing groups, to hydrogen atom transfer-based antioxidant activity as well [45]. Therefore, the MAAs palythine, porphyra-334, and shinorine exhibit strong antioxidant potential that is mediated through distinct and complementary antioxidant mechanisms.

It should be noted that although the bromophenols isolated from *Symphyocladia latiuscula* (Table 1 and Figure 2) exhibited remarkable antioxidant capacity [52], its activity has been evaluated using only a single antioxidant method. Consequently, further evaluation using complementary assays is required to comprehensively characterize its antioxidant profile.

Moreover, sulfated polysaccharides extracted from the red seaweeds *Gelidium pristoides* and *Gracilaria gracilis* enhanced CAT and SOD activities and elevated GSH levels in Zn-stimulated hippocampal neuronal cells [69]. In addition, in H_2_O_2_-stimulated RAW 264.7 cells, sulfated polysaccharides extracted from *Porphyra haitanensis* reduced the levels of ROS and malondialdehyde (MDA), a biomarker of lipid peroxidation, and enhanced the activity of antioxidant enzymes SOD, CAT and GPXs [50]. In a similar study, Marques et al. [65] reported that an exposure of 3T3-L1 pre-adipocytes to a sulfated polysaccharide fraction from *Gracilaria caudata* counteracted H_2_O_2_-induced oxidative stress, as evidenced by decreased ROS and MDA accumulation alongside the recovery of GSH levels and SOD activity. Also, oligosaccharides extracted from *P. haitanensis* (0.3 mg mL^−1^) enhanced intracellular SOD activity by 48.2%, as determined by a colorimetric assay, while reducing ROS levels by 15.1%, as assessed using a fluorometric method [70].

The antioxidant activity of dietary compounds, particularly in reducing NO levels released by macrophages, is also recognized as contributing to their anti-inflammatory effects. It is important to note that this anti-inflammatory activity may arise from modulating the synthesis and/or release of NO by macrophages rather than from direct scavenging alone [6,51,71]. Some dietary compounds may even act as immunostimulants, increasing NO production to enhance the immune response. This complex mechanism aims to optimize the inflammatory response, promoting immune efficiency while maintaining a balance between adequate defense and preventing excessive inflammation [6,72]. The immunomodulatory effects of dietary compounds can also be assessed by their impact on other key inflammatory mediators, including pro-inflammatory cytokines such as IL-6 and TNF-α and the transcription factor NF-κB, as well as enzymes such as iNOS and COX-2 [39,48,71,73].

Bioactive compounds obtained from red seaweeds demonstrated diverse modulatory effects on inflammatory processes. Sulfated polysaccharides extracted from the red seaweeds *G. pristoides* and *G. gracilis* reduced NO production in Zn-stimulated hippocampal neuronal cells, as determined by Griess-based nitrite assay, by approximately 17 and 25% (relative to the positive control), respectively, at 0.8 mg mL^−1^ [69]. In LPS-stimulated murine macrophages (RAW 264.7), porphyran extracted from discolored *Porphyra yezoensis* exhibited marked anti-inflammatory activity [51]. Nitric oxide production, quantified by the Griess-based nitrite assay, was reduced by approximately 87.5% relative to the positive control at a concentration of 1 mg mL^−1^ [51]. Consistently, reverse transcription-polymerase chain reaction (RT-PCR) and Western blot analyses revealed a pronounced downregulation of iNOS expression at both the transcriptional and translational levels, respectively, with near-complete inhibition observed at the same concentration. Furthermore, enzyme-linked immunosorbent assay (ELISA) demonstrated a significant suppression of TNF-α secretion [51]. Similarly, carrageenan fractions derived from several red seaweeds reduced NO production in LPS-stimulated RAW 264.7 macrophages [74]. Of these, the strongest effect was observed for a fraction composed of a mixture of carrageenan subtypes, which inhibited NO release by approximately 65% [74]. Furthermore, oligosaccharides isolated from *P. haitanensis* significantly attenuated inflammatory responses in LPS-stimulated IEC-6 cells. At 0.3 mg mL^−1^, NO production was reduced by approximately 50%, as determined by the Griess assay, while the expression of TNF-α, IL-6 and IL-1β was reduced by about 24–29% [70]. In another study, one terpenoid isolated from *K. alvarezii* exhibited in vitro inhibition of the pro-inflammatory enzyme 5-lipoxygenase (5-LOX) (IC_50_ = 1.04 mg mL^−1^) [53]. MAAs also showed anti-inflammatory potential. Becker et al. [73] observed that porphyra-334, isolated from *Porphyra* sp., attenuated NF-κB activation (~6% decline relative to control) in LPS-stimulated THP-1-Blue cells (cells specifically designed to monitor the NF-κB pathway). The suppression of NF-κB activation was measured by the decrease in embryonic alkaline phosphatase (SEAP) activity [73].

Conversely, porphyra-334, as well as shinorine, increased NF-κB activity (~5 and 34% increase relative to control, respectively) in unstimulated THP-1-Blue cells in Becker et al. [73], and since NF-κB activation is a key step in the inflammatory pathway, this response leads to enhanced iNOS expression and subsequent NO production, as well as increased levels of pro-inflammatory cytokines such as TNF-α and IL-6, thereby demonstrating immunostimulatory capacity [27]. Overall, Becker et al. [73] showed that different MAAs, namely, shinorine and porphyra-334, exhibit disparate immunomodulatory capabilities. In another study, Álvarez-Gómez et al. [39] reported that aqueous extracts from several red seaweeds significantly increased the secretion of IL-6 and TNF-α in RAW 264.7 cells, as quantified by ELISA, with levels approximately 4- to 41-fold and 12-fold higher than the control, respectively. Although MAAs were implicated as primary contributors to this immunostimulatory effect, the involvement of high-molecular-weight polysaccharides was also proposed [39]. Supporting this, sulfated polysaccharides extracted from red seaweeds were found to upregulate the production of iNOS, TNF-α and IL-6, alongside increased transcription of the corresponding genes in unstimulated RAW 264.7 cells [48,50,75]. Using the Griess method, an increase in NO production was observed in RAW 264.7 cells exposed to polysaccharides extracted from *P. haitanensis* [50]. Notably, the highest polysaccharide concentration (0.4 mg mL^−1^) induced higher NO levels than the positive control (LPS-stimulated cells) [50]. In addition, sulfated polysaccharides purified from *Porphyra umbilicalis* significantly increased TNF-α production and, to a lesser extent, IL-6 production, as quantified by ELISA [48]. It was noted that a commercially available R-phycoerythrin stimulated IL-6 and -8 production in THP-1 cells, as quantified by ELISA [76].

Overall, the dual anti-inflammatory and immunostimulatory effects observed for sulfated polysaccharides and MAAs (viz., porphyra-334) can be explained by the context-dependent modulation of immune signaling pathways. Under pro-inflammatory stimulation, these compounds attenuate excessive inflammatory responses by suppressing NF-κB activation and reducing iNOS expression, resulting in decreased NO and pro-inflammatory cytokine production [51,69,73]. Conversely, in unstimulated immune cells, these compounds promote controlled activation of NF-κB pathway, leading to moderate increases in NO and cytokine release consistent with immune activation rather than inflammation [48,50,73]. In MAAs, immunomodulatory activity is mainly associated with the imine-containing chromophore (Figure 2), whereas in sulfated polysaccharides, sulfate ester groups are regarded as primary determinants of immune activity, with molecular size and sulfation pattern shaping the final biological effect [9,73,77].

## 3. The Impact of Red Seaweed Compounds on Neuroprotection

### 3.1. Main Physiological Functions of Acetylcholine

**Figure 3:**
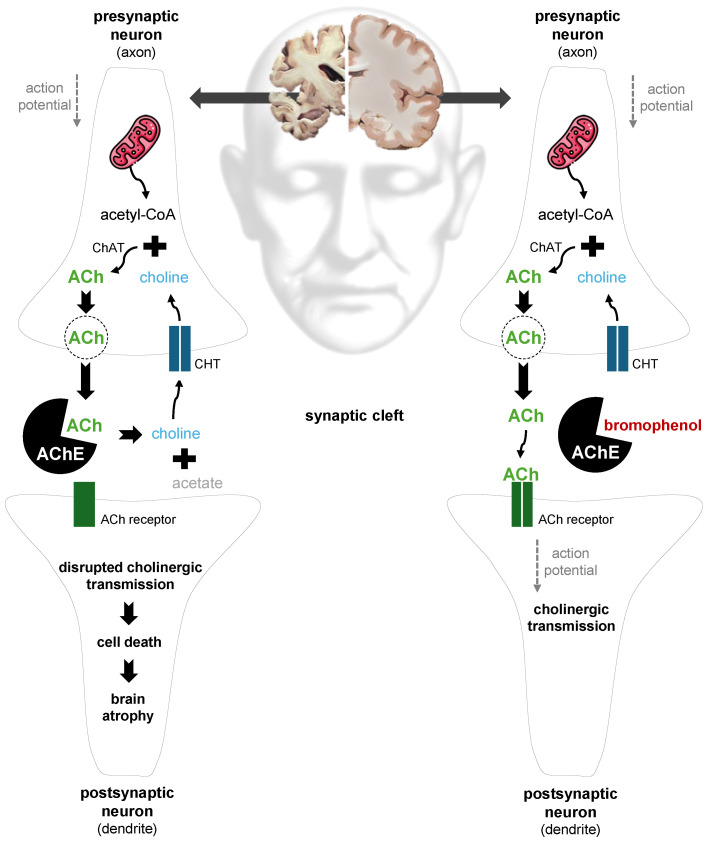
Central role of acetylcholine (ACh) in cholinergic neurotransmission and the potential modulatory mechanism by the bromophenol bis-(2,3,6-tribromo-4,5-dihydroxybenzyl) ether isolated from red seaweeds. ACh is synthesized in the presynaptic neuron from choline and acetyl-coenzyme A (acetyl-CoA). Choline enters the presynaptic neuron through the choline transporter (CHT), while acetyl-CoA is generated in mitochondria. The enzyme choline acetyltransferase (ChAT) catalyzes the synthesis of ACh. Once produced, ACh is stored in vesicles and released into the synaptic cleft in response to an action potential [80]. In the left synapse of the figure, the increased activity of acetylcholinesterase (AChE) in the synaptic cleft leads to the rapid hydrolysis of ACh into choline and acetate, stopping ACh from binding to its receptors on the postsynaptic neuron and impairing postsynaptic signaling. Choline can be reabsorbed by the presynaptic neuron [79]. This disruption of cholinergic transmission contributes to cell death, brain atrophy and the symptoms associated with Alzheimer’s disease (AD) [79,81]. In contrast, the right synapse in the figure illustrates the potential mechanism of action of the bromophenol bis-(2,3,6-tribromo-4,5-dihydroxybenzyl) ether with AChE [82]. The bromophenol binds to AChE, inhibiting the rapid degradation of ACh following its release from the presynaptic neuron. This inhibition allows ACh to bind to receptors on the postsynaptic cell, facilitating the continuation of the action potential. This sustained cholinergic neurotransmission can delay the progression of AD by reducing cell death and, consequently, mitigating brain atrophy [63,83]. While the figure is presented in a simplified and illustrative manner, it is essential to recognize that AD is a complex disorder influenced by a range of physiological alterations [81,84]. Abbreviations: acetyl-CoA, acetyl-coenzyme A; ChAT, choline acetyltransferase; ACh, acetylcholine; CHT, choline transporter; AChE, acetylcholinesterase.

Acetylcholine (ACh) is a key neurotransmitter in both the central and peripheral nervous systems of the human body (Figure 3). In the peripheral nervous system (PNS), it mediates signal transmission at the neuromuscular junction and within the autonomic nervous system. In the central nervous system (CNS), ACh primarily acts as a modulatory neurotransmitter, regulating neuronal excitability and synaptic plasticity, and thereby influencing essential cognitive processes such as memory, learning and attention [78,79,80].

ACh is synthesized in presynaptic neurons from two precursors: choline and acetyl-coenzyme A (acetyl-CoA) (Figure 3). Acetyl-CoA, generated in mitochondria, is transported into the cytoplasm, whereas choline, an essential dietary nutrient, enters neurons via a high-affinity choline transporter (CHT) located in the plasma membrane (Figure 3). In the axon terminal, ACh is produced in a single enzymatic step catalyzed by choline acetyltransferase (ChAT) and stored in synaptic vesicles until release [80,85]. Upon the arrival of an action potential, ACh is released into the synaptic cleft, where it diffuses and binds to specific receptors on the dendrite of a postsynaptic neuron, initiating a response in the receiving cell. Following receptor activation, ACh is rapidly hydrolyzed by acetylcholinesterase (AChE) into choline and acetate, terminating the action of ACh. Choline is then recycled via CHT for subsequent ACh synthesis, ensuring efficient and sustained neurotransmission within neural networks [78,80,85].

### 3.2. Red Seaweed-Derived Compounds as Acetylcholinesterase Inhibitors and Antioxidant/Anti-Inflammatory Agents in Alzheimer’s Disease

Neurodegenerative disorders are characterized by the progressive loss of neurons in specific regions of the nervous system, with Alzheimer’s disease (AD) being the most prevalent form [79,83]. AD is an irreversible disorder that impairs CNS function, progressively eroding memory, cognition and eventually basic tasks such as speech [81,86]. Aging is the greatest risk factor for its development [87,88]. The onset and progression of AD involves multiple pathological events, including aggregation of amyloid β (Aβ) peptides into plaques, the formation of intracellular neurofibrillary tangles by hyperphosphorylated tau proteins, neuroinflammation, and oxidative and nitrosative stress [81,83,84,89]. Collectively, these pathological changes contribute to cholinergic dysfunction, progressive neuronal loss (neurodegeneration) and the subsequent reduction in brain volume (atrophy) (Figure 3), particularly affecting the hippocampus, which is closely linked to the cognitive impairments characteristic of AD [83]. Although the precise etiology of AD remains unclear, likely involving a combination of genetic and environmental factors, research has focused heavily on increasing ACh levels by inhibiting AChE activity (Figure 3) [81,88,90].

AChE is predominantly localized in the synaptic cleft and neuronal membranes of the cholinergic system (Figure 3) [83]. Its main function is to catalyze the hydrolysis of ACh into choline and acetate (Figure 3) [79,91]. Inhibitors of AChE can delay the progression of AD, providing neuroprotective benefits [79,91]. Galantamine, a naturally occurring alkaloid extracted from plants in the Amaryllidaceae family, is a reversible, competitive AChE inhibitor clinically prescribed to alleviate cognitive decline in AD [81,83]. The primary mechanism of galantamine involves binding to specific sites within AChE, particularly the peripheral and anionic sites within the gorge, thereby reducing ACh hydrolysis (Figure 3) [63,83]. As a result, ACh levels rise in synaptic clefts, enhancing cholinergic signaling and supporting memory and cognition. Alongside galantamine, donepezil (a synthetic compound) and rivastigmine (a semisynthetic alkaloid) represent the three most prescribed therapeutics for AD, all acting as AChE inhibitors with distinct properties [83,87].

Numerous studies have concentrated on discovering novel natural neuroprotective agents derived from marine resources [92,93]. Recent review articles have highlighted the preventive and therapeutic potential of red seaweeds (as crude extracts, fractions or isolated compounds) in relation to AD, including Ghoran and Kijjoa [81], Pereira and Valado [90,94], Menaa et al. [79] and Olasehinde et al. [87]. Nonetheless, there is a need for a literature review on the AChE-inhibitory effects of red seaweed-derived compounds evaluated using Ellman’s method. Notably, sulfated polysaccharides [49,63,64,69], phenolic compounds (Figure 3) [54,82] and terpenoids [95,96] extracted from red seaweeds demonstrated significant AChE-inhibitory effects when evaluated using Ellman’s method (Table 2). This in vitro assay is used for assessing the hydrolysis of ACh by AChE, being commonly used to investigate the inhibitory potential of plant-derived metabolites on AChE activity [13,81]. In general, the effects of algal metabolites are likely mediated through reversible interactions with specific binding sites in AChE (Figure 3), resembling the mode of action of galantamine [63,81,97,98].

Three bromophenols isolated from *Symphyocladia latiuscula*, namely, bis-(2,3,6-tribromo-4,5-dihydroxybenzyl) ether, 2,3,6-tribromo-4,5-dihydroxybenzyl methyl ether (Figure 2 and Figure 3) and 2,3,6-tribromo-4,5-dihydroxybenzyl alcohol, were reported to exhibit the strongest AChE-inhibitory activity among red seaweed-derived compounds (Table 2) [82]. Notably, bis-(2,3,6-tribromo-4,5-dihydroxybenzyl) ether and 2,3,6-tribromo-4,5-dihydroxybenzyl methyl ether also displayed remarkable DPPH^•^ radical scavenging potential, as discussed previously (Table 1 and Figure 2) [52]. The authors further reported that replacement of the C7 side chain of the 2,3,6-tribromo-4,5-dihydroxybenzyl methyl ether with another 2,3,6-tribromo-4,5-dihydroxyl methyl ether moiety, connected by an *O*-linkage, resulting in bis-(2,3,6-tribromo-4,5-dihydroxybenzyl) ether, enhanced its inhibitory activity against AChE (Table 2 and Figure 2 and Figure 3) [82]. Molecular docking analysis indicated that these bromophenols interact with the AChE active site, with phenolic hydroxyl groups mediating interactions at the catalytic site, while the aromatic, brominated framework engages residues of the peripheral anionic site (Figure 2 and Figure 3) [82].

**Table 2:** Isolated compounds and fractions extracted from red seaweeds and their AChE-inhibitory capacity assessed using the in vitro assay based on Ellman’s method.

Seaweed Species	Isolated Compound/Fraction	Concentration	Result (% Inhibition)	Reference
*Symphyocladia latiuscula*	Bromophenols			
	bis-(2,3,6-tribromo-4,5- dihydroxybenzyl) ether (C_14_H_8_Br_6_O_5_)	0.002 mg mL^−1^	50%	[82]
	2,3,6-tribromo-4,5- dihydroxybenzyl alcohol (C_7_H_5_Br_3_O_3_)	0.003 mg mL^−1^	50%	[82]
	2,3,6-tribromo-4,5- dihydroxybenzyl methyl ether (C_8_H_7_Br_3_O_3_)	0.004 mg mL^−1^	50%	[82]
*Grateloupia elliptica*	Phlorotannin	0.033 mg mL^−1^	50%	[54]
	6,6′-bis(3,5-dihydroxyphenoxy)-1,1′-bioxanthrene-2,2′,4,4′,7,7′,9,9′-octol (6,6′-Bieckol; C_36_H_22_O_18_)			
*Laurencia dendroidea*	Halogenated sesquiterpenes			
	(3S,4R,6R)-4-bromo-10-chloro-5,5,9-trimethyl-1-methylidenespiro[5.5]undec-9-en-3-ol ((−)-elatol; C_15_H_22_BrClO)	0.100 mg mL^−1^	79%	[95]
	(2S,3R,6S,8S,9R)-2-bromo-8-chloro-1,1,9-trimethyl-5-methylidenespiro[5.5]undecane-3,9-diol ((−)-dendroidiol; C_15_H_24_BrClO_2_)	0.106 mg mL^−1^	73%	[95]
	(2S,3R,6S)-2,8-dibromo-9-chloro-1,1,9-trimethyl-5-methylidenespiro[5.5]undecan-3-ol ((−)-cartilagineol; C_15_H_23_Br_2_ClO)	0.124 mg mL^−1^	61%	[95]
*Laurencia johnstonii*	4-bromo-2-[(1S,2R,5R)-1,2-dimethyl-2-bicyclo[3.1.0]hexanyl]-5-methylphenol (laurinterol; C_15_H_19_BrO)	0.059 mg mL^−1 a^	50%	[96]
		2 mg mL^−1 a^	78%	[96]
*Asparagopsis armata*	Sulfated polysaccharides	0.3 mg mL^−1 b^	36%	[49]
		1 mg mL^−1 b^	~60%	[49]
		10 mg mL^−1 b^	90%	[49]
*Gelidium pristoides*		0.1 mg mL^−1 c^	~36%	[63]
		0.13 mg mL^−1 c^	~48%	[63]
		0.8 mg mL^−1^	~32%	[69]
*Gracilaria gracilis*		0.1 mg mL^−1 d^	~37%	[64]
		0.13 mg mL^−1 d^	~50%	[64]
		0.8 mg mL^−1^	~25%	[69]

^a^, ^b^, ^c^, ^d^ identical letters indicate the same compound/fraction tested at different concentrations. In Olasehinde et al. [69], Ellman’s method was applied to cell homogenates of Zn-stimulated hippocampal neuronal cells.

Another phenolic compound, the phlorotannin 6,6′-bieckol, also exhibited AChE-inhibitory activity [54]. However, its effect was less pronounced than that observed for bromophenols (Table 2), and no mechanistic explanation for its bioactivity was provided by the authors. Halogenated sesquiterpenes isolated from *Laurencia* spp., namely, laurinterol, (−)-elatol, (−)-dendroidiol and (−)-cartilagineol, demonstrated good potential to inhibit AChE activity, although they were less powerful than phenolic compounds (Table 2) [95,96]. In particular, molecular docking of (−)-elatol revealed interactions with key residues along the AChE active site gorge, including Trp86 located in the active site and Trp286 positioned in the peripheral anionic site, where interactions involving Br and Cl may contribute to the observed inhibitory activity [95].

Fractions of sulfated polysaccharides also demonstrated AChE-inhibitory activity, which varied depending on the seaweed species and the methodological approach used. However, these fractions, composed of heterogeneous mixtures of polysaccharides, generally exhibited lower inhibitory potential than previously reported purified compounds (Table 2). Sulfated polysaccharides isolated from *Asparagopsis armata* displayed an IC_50_ of approximately 0.70 mg mL^−1^, as reported by Feki et al. [49]. For *G. pristoides,* a lower IC_50_ value (~0.13 mg mL^−1^) was reported by Olasehinde et al. [63], whereas a substantially higher value (>0.8 mg mL^−1^) was observed in another study by the same authors [69]. A similar pattern was reported for *G. gracilis*, with an IC_50_ of 0.13 mg mL^−1^ [64] and a value exceeding 0.8 mg mL^−1^ under different experimental conditions [69]. In Olasehinde et al. [69], Ellman’s method was applied to cell homogenates of Zn-stimulated hippocampal neuronal cells, resulting in AChE inhibition, although to a lesser extent than that observed in cell-free assays and without Zn stimulation [63,64]. The inhibitory effect of sulfated polysaccharides may be attributed to strong interactions with positively charged residues within the AChE gorge, particularly at the peripheral anionic site and the anionic subsite [49,63,69]. These interactions are potentially mediated by sulfate ester groups (–OSO_3_^−^), which promote enzyme binding, thus hindering substrate access [64].

Additionally, Machado et al. [98] reported that the crude extract of *Ochtodes secundiramea* exhibited 48% AChE inhibition at a concentration of 0.4 mg mL^−1^, as determined by Ellman’s method. Subsequent activity-guided thin-layer chromatography (TLC) followed by gas chromatography–mass spectrometry (GC-MS) analysis revealed that the bioactive fraction was composed exclusively of halogenated monoterpenes [98]. Specifically, bromine-containing groups in brominated monoterpenes may promote enzyme inhibition through strong interactions with electron-rich functional groups of amino acid residues within the AChE active site [98]. Syad et al. [99] reported that seven fractions obtained from the benzene extract of *Gelidiella acerosa* by column chromatography exhibited significant AChE-inhibitory activity (~25 to 45% at 0.2 mg mL^−1^), and that liquid chromatography—mass spectrometry (LC-MS), high-performance thin-layer chromatography (HPTLC) and molecular docking analyses identified phytol, an acyclic diterpene alcohol, as a major constituent of the active fractions and confirmed its key contribution to the observed bioactivity. Subsequently, Syad et al. [99] evaluated a commercial phytol using Ellman’s method and reported a low IC_50_ value (0.003 mg mL^−1^), indicating potent AChE inhibition that is comparable to that observed for bromophenols isolated from red seaweeds present in Table 2. Furthermore, molecular docking analyses suggested that phytol interacts with AChE through arginine residues within the enzyme’s active site.

Furthermore, neurons within the CNS are particularly susceptible to excessive ROS generation due to their high O_2_ consumption and energy demands [84,89]. Mitochondrial dysfunction, a central mediator of oxidative stress, induces oxidative damage that accelerates apoptosis and brain aging, significantly contributing to AD progression [63,84,88,100]. In addition, oxidative and nitrosative stress can trigger acute neuroinflammatory responses aimed at restoring homeostasis. However, when such responses persist, chronic neuroinflammation activates cascading processes that progressively damage neurons [79,88,94]. Accordingly, dietary strategies incorporating functional foods and nutraceuticals rich in bioactive molecules exhibit promising potential in managing the early stages of AD [79,88,94]. Bioactive compounds from red seaweeds have been considered for their potential in mitigating oxidative and nitrosative stress that cause damage to neurons in AD [69,79,87,94]. Specifically, as presented before, in a comprehensive investigation, Olasehinde et al. [69] reported that sulfated polysaccharides extracted from *G. pristoides* and *G. gracilis* enhanced SOD and CAT activities, elevated GSH levels, reduced NO production and exhibited AChE-inhibitory activity (Table 2) in Zn-stimulated hippocampal neuronal cells, thereby highlighting their potential for protecting neuronal cells against pathological processes associated with AD.

## 4. Conclusions and Future Directions

Oxidative and nitrosative stress are multifactorial processes implicated in the onset and progression of numerous diseases, including cancer, chronic inflammatory disorders and Alzheimer’s disease. Cholinergic dysfunction is likewise a characteristic feature in the development of Alzheimer’s disease. In vitro bioactivity testing remains indispensable for evaluating isolated compounds and fractions obtained from red seaweed biomass, as it allows the detailed investigation of their specific biochemical and cellular mechanisms of action. Such approaches include chemical assays for evaluating antioxidant activity (e.g., DPPH^•^ and ABTS^•+^ assays), biochemical assays for assessing neuroprotective potential (e.g., acetylcholinesterase inhibition using Ellman’s method) and cell-based assays for investigating immunomodulatory effects (e.g., NO production in RAW 264.7 macrophages). Therefore, red seaweed compounds, particularly sulfated polysaccharides, oligosaccharides, MAAs, phycoerythrin, bromophenols, phlorotannin and terpenoid-derived metabolites, occur as promising bioactive molecules exhibiting in vitro antioxidant, anti-inflammatory, immunostimulatory and acetylcholinesterase inhibitory activities, mediated through multiple regulatory mechanisms.

Ongoing advances in this field could contribute to a better understanding of the preventive and therapeutic potential of red seaweeds in complex illnesses such as cancer, chronic inflammatory disorders and Alzheimer’s disease. For example, substantial gaps remain in the current literature regarding red seaweed-derived bioactive compounds as AChE inhibitors. To date, direct evidence supporting the AChE-inhibitory activity of MAAs is lacking, despite their well-documented antioxidant and immunomodulatory properties, highlighting the need for targeted investigations in this area. Considering the potential demonstrated, bromophenols should be further explored regarding their possible role as natural agents for neurodegenerative diseases. Furthermore, additional studies are required to elucidate which specific subtypes of sulfated polysaccharides exhibit the greatest bioactive potential, as most reported studies focus on polysaccharide fractions rather than isolated subtypes of carrageenan or agar. Future research should also focus on in vivo and clinical studies to validate the bioactivities of red seaweed metabolites under physiological conditions. Studies addressing bioavailability, metabolism and potential synergistic interactions among seaweed-derived molecules are crucial to support their uses in human nutrition as functional foods and nutraceuticals.

## Data Availability

No new data were created or analyzed in this study. Data sharing is not applicable to this article.
